# Metabolic and clinical responses to Bunium Persicum (black caraway) supplementation in overweight and obese patients with type 2 diabetes: a double-blind, randomized placebo-controlled clinical trial

**DOI:** 10.1186/s12986-020-00494-4

**Published:** 2020-08-26

**Authors:** Saber Jafari-Maskouni, Mansour Shahraki, Milad Daneshi-Maskooni, Alireza Dashipour, Ali Shamsi-Goushki, Zinat Mortazavi

**Affiliations:** 1 Department of Nutrition, School of Medicine, Zahedan University of Medical Sciences, Zahedan, Iran; 2 Department of Nutrition, School of Medicine, Jiroft University of Medical Sciences, Jiroft, Iran; 3 Department of Food Science and Nutrition, Cellular and Molecular Research Center, Department of Clinical Biochemistry, Zahedan University of Medical Sciences, Zahedan, Iran

**Keywords:** Type 2 diabetes, Bunium Persicum, Glucose indices, Lipids, Nesfatin-1

## Abstract

**Background:**

Diabetes mellitus is the most common metabolic disorder worldwide. We aimed to determine the metabolic and clinical responses to Bunium Persicum (Black Caraway) supplementation in overweight and obese patients with T2DM.

**Methods:**

Participant recruitment took place in the diabetic clinic of Bu-Ali hospital in Zahedan. Due to the eligibility criteria, 60 participants were randomly placed into two groups, namely placebo (*n* = 30) and BP (*n* = 30). The supplementation was considered one 1000 mg capsule 2 times /day BP by meals (lunch and dinner) for 8 weeks. Physical activity levels, dietary intakes, anthropometric measurements [weight, height, and waist circumference], glycemic indices [fasting blood glucose (FBG) and insulin (FBI)], blood lipids [triglyceride (TG), total cholesterol (TC), high-density lipoprotein cholesterol (HDL-c), and low-density lipoprotein cholesterol (LDL-c)], and serum nesfatin-1 level were determined. Homeostasis model assessment-insulin resistance (HOMA-IR), Quantitative insulin sensitivity checks index (QUICKI), and Body Mass Index (BMI) were computed.

**Results:**

In comparison with placebo, BP significantly decreased FBG, HOMA-IR, and BMI (*P <  0.05*). The differences in the FBI, QUICKI, TG, TC, LDL, HDL, WC, and Nesfatin-1 were not significant (*P > 0.05*).

**Conclusion:**

BP supplementation improved serum glucose indices and BMI among overweight and obese T2DM patients. Further trials are needed to confirm results.

**Trial registration:**

Iranian Registry of Clinical Trials (IRCT), IRCT20181207041876N1, Registered 18/01/2019, https://irct.ir/trial/35752

## Background

Diabetes is a multifactorial autoimmune disorder determined by a high blood glucose level. The prevalence of diabetes mellitus type 2 (T2DM) is globally increasing, and its complications are a significant health problem [1, 2]. In 2014, over 422 million had diabetes, and a continuous rise in DM prevalence was expected [3].

Being overweight, obesity, impaired blood glucose, and hyperlipidemia are some risk factors of T2DM [4, 5]. Some metabolic perturbations, such as B-cell dysfunction, impaired insulin secretion, insulin resistance, and dyslipidemia contribute to the pathogenesis of diabetes [6].

Adipose tissue is an active endocrine organ that produces adipokines that control physiological functions such as immunity, inflammation, and energy homeostasis [7]. Nesfatin-1 adipokine plays a role in regulating appetite and body fat storage by affecting glucose metabolism, phosphorylation of specific signaling proteins through AMP-activated protein kinase, and increasing liver insulin sensitivity [8, 9]. This adipokine is expressed in the parts of the brain that interfere with metabolism regulation and dietary behavior [10]. Expression of the nesfatin-1 gene is activated by peroxisome proliferator-activated receptors (PPARs), especially PPARγ [11, 12]. Nesfatin-1 can decrease food intake when administered into the third cerebral ventricle of rats [13]. Fasting blood nesfatin-1 was significantly reduced in T2DM patients compared to healthy subjects and might be one of the appetite-related hormones involved in diabetic Polyphagia [14].

The management of diabetes to prevent complexity includes the changes in dietary patterns, regular physical activity, and anti-diabetic medications [15, 16].

The various types of traditional, complementary, or alternative therapies have been increasingly utilized to treat diabetes in human and animal models of type 2 diabetes [17]. Herbal medicines are a significant part of these therapies [18]. One of the medicinal plants is Bunium Persicum (Black Caraway). Bunium Persicum which belongs to the Apiaceae family and is generally known in Iran as ‘Zireh Siah’ has always been used in traditional Iranian medicine for specific disorders [19, 20]. The main constituents of BP, which have been demonstrated in recent studies, are cumin aldehyde, caffeic acid, p-coumaric acid, gamma-terpinene, and p-cymene, cuminal, flavonoids (such as quercetin and kaempferol) and a high level of polyphenols compound [21–25]. The reported effects of cumin aldehyde, as one of the most studied agent, include the inhibition of aldose reductase and r-Glucosidase, the insulinotropic, and b cell-protective action, anti-obesity, and liver protective effect [26–29].

Considering the data available, previous studies on the effect of Bunium Persicum (Black Caraway) on T2DM patients and healthy subjects were inconsistent. Some previous animal and human studies indicated that caraway intake could improve lipid profiles and decrease blood glucose concentrations [30, 31]. However, Ghorbani et al. reported that caraway supplementation had no beneficial effects on fasting blood glucose concentrations. Some other researchers revealed no significant impact of caraway intake on lipid profiles and fasting blood glucose [31, 32].

Evidence has suggested that Bunium Persicum and *Carum carvi* are plant members of the Apiaceae family and have similar active ingredients [33, 34]. Besides, slimming, appetite-suppressing, hypoglycemic, and antihyperlipidemic effects of *Carum carvi* have been shown in recent studies [35, 36]. Considering food intake decreasing effects of nesfatin-1 demonstrated in previous studies [37], we hypothesized Bunium Persicum could suppress appetite and reduce food intake by increasing the levels of nesfatin-1 which can, in turn, lead to the decrease of blood glucose and lipid profiles. Also, no previous human studies have been conducted regarding the effects of BP on the metabolic and clinical markers and nesfatin-1 in overweight and obese T2DM patients. This trial was designed to assess the metabolic and clinical responses to Bunium Persicum (Black Caraway) supplementation in overweight and obese patients with type 2 diabetes.

## Methods

### Ethical considerations

This study was approved by the ethics committee of Zahedan University of Medical Sciences (IR.ZAUMS.REC.1397.332) and registered in the Iranian Registry of Clinical Trials (IRCT20181207041876N1) on 18/01/2019. The participants were T2DM patients with overweight and obesity referred to the diabetic clinic of Bu-Ali Hospital of Zahedan. This trial lasted from 23 June 2019 to 22 October 2019.

An informed written consent form provided by the subjects before data collection. Participants were informed at study’s outset about risks and possible side effects of BP, blood sampling, the confidentiality of participants’ personal information, right to withdraw from the study at any point, free-of-charge participation in the trial, and weekly contact by the researcher to participants, and approval section.

### Study design and subjects

The present double-blind, randomized, placebo-controlled clinical trial handled 60 overweight or obese patients with T2DM. they were randomly placed into two groups (Placebo group [*n* = 30] and Bunium Persicum group [n = 30]) by the block randomization method.

Selected patients took Bunium Persicum and Placebo capsules for 8 weeks. The packaging of supplements and capsules were similar in appearance. The researchers and subjects were blinded to group assignment and capsule content to the end of the analysis.

**Inclusion criteria** were T2DM diagnosed, utilizing oral hypoglycemic drugs, 30–65 years, and 25 ≤ BMI < 40 kg/m^2^. **Exclusion criteria** were psychiatric disorders, acute systemic disease, cystic fibrosis, muscular dystrophy, protein malnutrition, the history of gastrointestinal surgery, gastrointestinal disorders, disability, uncontrolled hypertension (> 140/90 mmHg), consumption of probiotics, multivitamin-mineral, and antioxidant supplements during the past 8 weeks, professional athlete, consumption of statins, antihypertensive, and caraway interacting drugs, pregnancy or lactation, the alcohol, cigarette, and drug abuse during the past 8 weeks, losing weight during the past 8 weeks, the change of type and dose of T2DM medications, and no consuming more than 10% of capsules.

The CONSORT guidelines for reporting randomized trials were checked, and a completed CONSORT checklist was added as an Additional file.

### Intervention and randomization

**Fig. 1:**
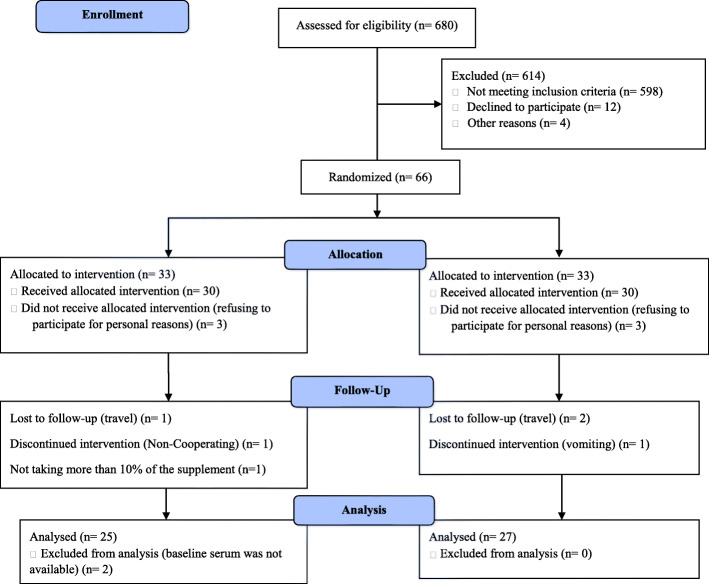
Flow chart of the study participants

The participants were distributed into 2 parallel groups by an assistant (BP [*n* = 30] or placebo [n = 30] groups) using block randomization method. We used a stratified randomization method for age and sex. The proportion of 1:1 was used between the BP and placebo groups. Three patients from the BP group and two patients from the placebo group refused to participate after randomization and before the beginning of the trial (Fig. 1).

The intervention allocation was blinded for investigators and participants as A and B packages. The Herbarium center of the Faculty of Pharmacy, Kerman University of Medical Sciences, Kerman, Iran made the BP and placebo capsules. The size, color, and shape of the capsules were similar. The content of capsules was 1 g of BP or Starch powder. Before the supplementation, the capsules were placed with each other for one week to smell similarly. The amount of absorbed Bunium Persicum volatile oil by the placebo capsules was meager to alter the health parameters. A previous study investigated the effect of cumin (one of the Apiaceae family plants) on overweight and obese women, the dose of supplements was recognized at 2 g per day or 2 capsules per day, taken with meals (twice a day with lunch and dinner) [38]. But, the consumption and absorption of BP with or without meals need to be further studied. The capsules were distributed monthly. The monitoring of participants’ compliance and the voice calls were done monthly and weekly, respectively. The duration of the intervention period was 8 weeks.

BP voucher number was Bunium Persicum (Boiss) B. Fedtch, Family: Apiaceae, KF1141–1. The analysis of BP was performed by the Herbarium center of Kerman Faculty of Pharmacy, Kerman University of Medical Sciences, Kerman, Iran. The gas chromatography-mass spectrometry (GC-MS) analysis showed that the main components of Bunium Persicum essential oils were 46.10% γ-Terpinene and 15.50% cumin aldehyde.

### Measurements and evaluations

#### Diet and physical activity

T2DM patients were selected according to the eligibility criteria. The details of the research were explained, and informed consent was taken by the principal researcher. The general questionnaire, 24-h food recall, and short-form International Physical Activity Questionnaire (SF-IPAQ) were used to determine the general characteristics, dietary intakes (at the beginning and end), and physical activity levels (at the beginning and end).

24-h food recall was valid and reliable in Iran [39]. The modified N4 (nutritionist IV) software was used to analyze the dietary intake status [39, 40].

The SF-IPAQ questionnaire includes 7 classified questions to determine physical activity levels (1–3 or low-to-high levels). It was valid and reliable in Iran [11, 41, 42].

#### Anthropometric measurements

We measured weight without shoes, with minimal clothing by 100-g accuracy (at the beginning and end), height without shoes, standing, heels sticking to the wall, flat and forward head, and with 0.5-cm accuracy (at the beginning), and waist circumference with minimal clothing, at the middle of the last rib and the iliac crest (at the beginning and end) using a digital scale and stadiometer (*Seca® Germany, Model: 7551021994*). Body mass index (BMI) was calculated by the following formula.

$$ \mathrm{BMI}={\mathrm{Weight}}_{\left(\mathrm{Kg}\right)}/{{\mathrm{Heigh}}^2}_{\left(\mathrm{Metere}\right)} $$

#### Blood biomarkers measurement

The peripheral venous blood samples (10 ml) were taken after 12-h overnight fasting and centrifuged for 20 min (3000 *g*). The day of determining fasting plasma glucose and blood taking was the same. The residual serums (5 cc) were frozen and kept at − 80^°C^ until the analysis.

Serum nesfatin and insulin were measured using the sandwich ELISA by an automatic device (*Elisys Uno Human®*) and specific kits (*Shanghai Crystal Day Biotech Co. Ltd®; Intra-assay CV < 8%, Inter-assay CV < 10*% and *diametra® Co of Italy, DCM076–8; Intra-assay CV ≤ 5%, Inter-assay CV ≤ 10%*, respectively). The measurement of FBG was done using the glucose oxidase method, the Hitachi analyzer device (*q17®*), and the specific kit (*Bionik®, Liquid Stable, Glucose oxidase GOD-POD, Mono-reagent; Intra-assay CV ≤ 2.10%, Inter-assay CV ≤ 3.09%*). Serum TC, TG, LDL-C, and HDL-C were determined using Hitachi analyzer device (*q17*®) and the specific kits (−*Bionik*®*, Liquid Stable, Enzymatic Colorimetric CHOD-POD*, −*Bionik*®*, Liquid Stable, GPO-POD, Mono-reagent*, −*Bionik*®*, Liquid Stable, Direct. Enzymatic Colorimetric*, and -*Bionik*®*, Liquid Stable, Direct. Enzymatic Colorimetric*, respectively) The intra- and inter-assay coefficients of variation for them were ≤ 1.22% and ≤ 6.90%, ≤1.57% and ≤ 7.70%, ≤1.76% and ≤ 0.65%, and ≤ 0.70% and ≤ 1.50%, respectively. HOMA-IR and QUICKI indices were calculated using FBG and FBI by the standard formulas [43].

### Sample size

According to the “two mean comparison formula” and a similar study [44], the sample size was determined, considering the cholesterol as the main variable, and errors I and II, the mean difference of cholesterol between the groups, the standard deviation of group 1 and 2 were 0.05 and 0.20, 17.38, 29.34 and 4.77 respectively.

$$ \mathrm{n}={\left({\mathrm{Z}}_{1\hbox{-} \upalpha /2}+{\mathrm{Z}}_{1\hbox{-} \upbeta}\right)}^2\left({{\mathrm{S}}_1}^2+{{\mathrm{S}}_2}^2\right)/{\left({\upmu}_1\hbox{-} {\upmu}_2\right)}^2 $$

Considering the anticipation of a 30% drop-out, the sample size was estimated at 30 participants in each group.

### Statistical analyses

The normal distribution of the data was checked by the Kolmogorov-Smirnov test. The baseline variables and dietary intakes of participants in the two groups were compared using t- and chi-square tests for quantitative and qualitative variables, respectively. We used a two-way repeated-measure analysis of variance (TWRM-ANOVA) to assess time effects and time by treatment interaction effects on all dependent variables and adjust it for dietary intake of vitamin B12. *P*-value < 0.05 was statistically considered significant. The statistical analyses were done using SPSS software (Version 16; SPSS Inc., Chicago, IL). The final dataset was only accessible to the principal researcher. The research results were presented using the publication.

## Results

### General characteristics

According to the medical history, from June to July of 2019, the screening of 680 patients was done and 82 of them were eligible for the participating. Then, 12 patients declined and 4 patients could not participate. Furthermore, 66 participants were randomized, with 3 participants in the BP group and 3 participants in the placebo group refusing to participate and did not receive the capsules. Thus, 60 participants (Bunium Persicum *n* = 30; placebo n = 30) completed the first visit. Also, 6 patients could not continue the follow-up phase (for personal reasons, travel, and did not take more than 10% of the capsules; Bunium Persicum n = 3; placebo n = 3). Moreover, the baseline serum sample of 2 participants in the placebo group wasn’t available. Finally, data of 52 participants were analyzed (Fig. 1).

The general characteristics and status of physical activity and blood-sugar-lowering medications were presented in Table 1. The differences between the two groups weren’t statistically significant (Table 1). More than 95% of assigned capsules were well consumed in both groups.

**Table 1:** General characteristics and physical activity of overweight or obese patients with type 2 diabetes mellitus (T2DM)

General variables and physical activity		Caraway (*n* = 27) n(%) or Mean (SD)	Placebo (*n* = 25) n(%) or Mean (SD)	*P-value*
Age (yrs)		52.41(10.78)	50.09(10.87)	0.49^*^
Gender	male	8.00(29.62)	9.00(36.00)	0.62^**^
	female	19.00(70.38)	16.00(64.00)	
Blood sugar-lowering agents	Metformin	18.00(66.67)	16.00(64.00)	0.76^**^
	Glibenclamide	4.00(14.81)	5.00(20.00)	
	Metformin+Glibenclamide	4.00(14.81)	4.00(16.00)	
	other	1.00(3.70)	0.00(0.00)	
Education level	up to associate degree	11.00(40.74)	12.0(48.00)	0.60^**^
	Bachelor and higher	16.00(59.26)	13.0(52.00)	
Economic level	Low/moderate (≤6 living items)	8.00(29.62)	10.0(40.00)	0.43^**^
	High (≥7 living items)	19.0(70.38)	15.0(60.00)	
Physical activity level (Baseline)		1.42(0.57)	1.33(0.54)	0.59^*^
Physical activity level (End)		1.35(0.56)	1.43(0.51)	0.60^*^

*t-test, **Chi-square

### Dietary intakes and blood biomarkers

Differences in the blood biomarkers between the two groups at baseline were not statistically significant (*P < 0.05*, Table 2). The dietary intakes of the baseline were similar between groups except for carbohydrate (gr), protein (%), and iron, which were higher in the BP group (Table 3).

**Table 2:** Comparison of baseline mean for BMI and serum nesfatin-1, glucose indices, and lipid profile in overweight or obese patients with type 2 diabetes mellitus (T2DM)

Baseline Dependent Variables	Caraway (n = 27) n(%) or Mean (SD)	Placebo (n = 25) n(%) or Mean (SD)	P-value^*^
BMI (kg/m2)	28.77(3.77)	29.19(3.27)	*0.69*
WC (cm)	100.32(7.06)	100.35(8.45)	*0.94*
FBG (mg/dl)	175.38(69.89)	174.04(52.30)	*0.92*
FBI (μIU/ml)	9.39(6.77)	11.63(7.51)	*0.31*
HOMA-IR (score)	4.21(3.72)	4.98(3.11)	*0.47*
QUICKI (score)	0.45(0.01)	0.44(0.01)	*0.57*
Nesfatin-1 (ng/ml)	4.37(1.65)	5.46(2.14)	*0.07*
TC (mg/dl)	159.04(36.78)	152.62(48.96)	*0.61*
TG (mg/dl)	163.34(117.15)	164.23(82.37)	*0.97*
LDL-C (mg/dl)	81.34(27.61)	83.27(29.58)	*0.82*
HDL-C (mg/dl)	44.96(11.99)	42.95(7.65)	*0.51*

*t-test; **BMI**: body mass index, **WC**: waist circumference, **HOMA-IR**: homeostasis model assessment-insulin resistance, **QUICKI**: quantitative insulin sensitivity check index, **FBG**: fasting blood glucose, **FBI**: fasting blood insulin, **TC**: total cholesterol, **TG**: triglyceride, **HDL-C**: high-density lipoprotein cholesterol, **LDL-C**: low-density lipoprotein-cholesterol

**Table 3:** Mean of dietary intakes during the study on overweight or obese patients with type 2 diabetes mellitus (T2DM)

Dietary intakes during the study	Bunium Persicum (*n* = 27) Mean(95% CI)	Placebo (*n* = 25) Mean(95% CI)	*P-value*^$^
Energy (kcal)	1982.92(1951.61, 2014.24)	1799.37(1793.42, 1805.33)	*0.45*
Protein (g)	72.10(71.57, 72.64)	77.85(78.91, 76.79)	*0.24*
Protein (%)^*^	14.40(14.54, 14.27)	17.35(17.50, 17.21)	*0.76*
Carbohydrate (g)^*^	274.65(268.63, 280.67)	227.01(230.75, 223.27)	*0.27*
Carbohydrate (%)	54.81(54.49, 55.14)	49.63(50.44, 48.82)	*0.25*
Fat (g)	69.15(69.02, 69.29)	66.55(65.35, 67.76)	*0.55*
Fat (%)	30.78(30.96, 30.61)	33.03(31.99, 34.07)	*0.17*
Cholesterol (mg)	133.4(123.5, 143.3)	130.9(132.3, 129.5)	*0.11*
Saturated fat (g)	20.29(19.58, 21.01)	16.09(15.88, 16.30)	*0.69*
Monounsaturated fatty acid (g)	25.31(26.90, 23.72)	24.45(25.02, 23.88)	*0.42*
Polyunsaturated fatty acid (g)	16.98(16.42, 17.54)	17.84(17.71, 17.97)	*0.63*
Vitamin A [RAE] (μg)	806.25(884.32, 728.18)	675.98(752.71, 599.26)	*0.99*
Vitamin C (mg)	136.41(123.35, 149.47)	125.41(118.22, 132.60)	*0.59*
Potassium	2708.64(2624.96, 2792.33)	2588.11(2540.45, 2635.78)	*0.81*
Calcium (mg)	823.44(837.12, 809.77)	911.81(934.20, 889.42)	*0.88*
Iron (mg)^*^	19.42(19.15, 19.69)	15.25(15.23, 15.28)	*0.40*
Vitamin D (μg)	0.61(0.49, 0.73)	0.52(0.44, 0.60)	*0.81*
Vitamin E (mg)	17.54(19.75, 15.34)	16.02(16.61, 15.42)	*0.18*
Vitamin B1 (mg)	1.89(2.13, 1.66)	1.85(1.88, 1.83)	*0.20*
Vitamin B2 (mg)	1.82(1.90, 1.74)	1.78(1.86, 1.71)	*0.88*
Vitamin B3 (mg)	23.81(23.89, 21.74)	25.75(25.60, 25.91)	*0.33*
Vitamin B6 (mg)	2.48(2.54, 2.42)	2.84(3.27, 2.41)	*0.37*
Folate (DFE) (μg)	143.69(138.29, 149.09)	144.68(147.47, 141.89)	*0.51*
Vitamin B12 (μg)	2.41(2.30, 2.52)	2.21(2.39, 2.03)	*0.03*
Vitamin K (μg)	181.98(129.29, 234.67)	154.90(106.78, 203.03)	*0.91*
Zinc (mg)	9.54(9.51, 9.58)	8.58(8.39, 8.77)	*0.70*
Selenium (μg)	33.15(32.25, 34.06)	39.18(38.96, 39.40)	*0.82*
Total fiber (g)	4.69(4.43, 4.96)	4.15(3.76, 4.55)	*0.42*

*The dietary intakes at the baseline were higher in the BP group (P <  0.05)

$Two way repeated measures-ANOVA (TWRM-ANOVA)

The dietary intakes were approximately similar in two groups during the trial except for vitamin B12, which was higher in the BP group (*P* <  0.05, Table 3). So, it was entered as a confounding factor in the final analysis model. The mean differences of TG, TC, LDL, and HDL weren’t statistically significant within the BP group (*P* > 0.05). On the other hand, nesfatin-1 and QUICKI increased and FBG, FBI, HOMA-IR, BMI, and WC decreased significantly (*P* <  0.05). The mean differences of glucose indices (FBS, FBI, HOMA-IR, QUICKI), lipid profiles (TC, TG, LDL-C, HDL-C), and nesfatin-1 weren’t statistically significant within the placebo group (*P* > 0.05) (Table 4).

**Table 4:** The changes in BMI, WC, serum nesfatin-1, glucose indices, and lipid profile in overweight or obese patients with type 2 diabetes mellitus (T2DM)

Variables	Supplement	Baseline Mean	End Mean	*P-value*^*$*^	Mean Changes (95% CI)	*P-value*^#^		
						Time	Treatment	Interaction
BMI	Caraway (n = 27)	28.77(3.77)	28.32(4.06)	< 0.01	−0.45 (0.16, 0.72)	*0.43*	*0.48*	*0.02*
	Placebo (n = 25)	29.19(3.27)	29.42(3.28)	0.38	0.23 (−.80, 0.30)	*0.83*	*0.54*	*0.04*
WC (cm)	Caraway (n = 27)	100.32(7.06)	99.04(7.41)	< 0.01	−1.28 (0.49, 2.06)	*0.01*	*0.79*	*0.04*
	Placebo (n = 25)	100.35(8.45)	100.21(8.29)	0.73	−0.13 (−0.67, 0.94)	*0.22*	*0.60*	*0.12*
FBG (mg/dl)	Caraway (n = 27)	175.38(69.89)	142.65(53.84)	< 0.01	−32.73 (13.67, 51.79)	*0.04*	*0.32*	*< 0.01*
	Placebo (n = 25)	174.04(52.30)	178.95(73.49)	0.57	4.90 (−22.70, 12.90)	*0.16*	*0.82*	*0.02*
FBI (μIU/ml)	Caraway (n = 27)	9.39(6.77)	5.59(2.50)	0.03	−3.79 (0.33, 7.25)	*0.24*	*0.01*	*0.15*
	Placebo (n = 25)	11.63(7.51)	12.03(8.98)	0.87	0.40 (−5.35, 4.55)	*0.14*	*0.01*	*0.05*
HOMA-IR	Caraway (n = 27)	4.21(3.72)	2.27(1.32)	0.04	−1.94 (0.91, 3.78)	*0.18*	*0.01*	*0.04*
	Placebo (n = 25)	4.98(3.11)	5.16(4.68)	0.87	0.18 (−2.48, 2.12)	*0.17*	*0.03*	*0.02*
QUICKI	Caraway (n = 27)	0.45(0.01)	0.47(0.03)	0.01	0.02 (−.03, 0.0)	*0.01*	*0.13*	*0.03*
	Placebo (n = 25)	0.44(0.01)	0.45(0.03)	0.76	0.01 (−.04, 0.1)	*0.05*	*0.43*	*0.05*
TC (mg/dl)	Caraway (n = 27)	159.04(36.78)	159.50(37.06)	0.94	0.46 (−13.34, 12.42)	*0.45*	*0.79*	*0.51*
	Placebo (n = 25)	152.62(48.96)	159.95(45.22)	0.4	7.33 (−24.92, 10.26)	*0.88*	*0.57*	*0.93*
TG (mg/dl)	Caraway (n = 27)	163.34(117.15)	175.27(121.37)	0.39	−11.92 (−39.92, 16.08)	*0.14*	*0.94*	*0.86*
	Placebo (n = 25)	164.23(82.37)	179.29(100.35)	0.19	15.04 (−38.13, 8.03)	*0.26*	*0.92*	*0.82*
LDL-C (mg/dl)	Caraway (n = 27)	81.34(27.61)	82.57(27.67)	0.84	1.22 (−13.74, 11.29)	*0.64*	*0.86*	*0.46*
	Placebo (n = 25)	83.27(29.58)	77.78(39.33)	0.42	−5.49 (−8.50, 19.49)	*0.94*	*0.48*	*0.12*
HDL-C (mg/dl)	Caraway (n = 27)	44.96(11.99)	44.19(11.22)	0.52	−0.77 (−1.66, 3.20)	*0.89*	*0.70*	*0.35*
	Placebo (n = 25)	42.95(7.65)	44.00(9.22)	0.52	1.04 (−4.36, 2.27)	*0.67*	*0.84*	*0.33*
Nesfatin-1 (ng/ml)	Caraway (n = 27)	4.37(1.65)	6.14(3.38)	0.02	1.77 (−3.24, 0.29)	*0.01*	*0.26*	*0.59*
	Placebo (n = 25)	5.46(2.14)	6.64(3.86)	0.17	1.18 (−2.90, 0.54)	*0.23*	*0.67*	*0.79*

**$**Paired t-test; **#**Two way repeated measures-ANOVA (TWRM-ANOVA), top row P-_value_: unadjusted; bottom row P-_value_: adjusted for vitamins B12 dietary intake

**BMI**: body mass index, **WC**: waist circumference, **HOMA-IR**: homeostasis model assessment-insulin resistance, **QUICKI**: quantitative insulin sensitivity check index, **FBG**: fasting blood glucose, **FBI**: fasting blood insulin, **TC**: total cholesterol, **TG**: triglyceride, **HDL-C**: high-density lipoprotein cholesterol, **LDL-C**: low-density lipoprotein-cholesterol

The final analysis model (time by treatment interaction effect) showed a significant decrease in FBG, HOMA-IR, BMI, WC, and a significant increase in the QUICKI in the BP group in comparison with the placebo group (*P* <  0.05) (Table 4). The differences were similar after adjustment for confounding factors (*P* <  0.05) with an exception for WC (*P* = 0.12) (Table 4).

### Side effects

Only one participant in the BP group reported vomiting.

## Discussion

It was the first time that the metabolic and clinical responses of Bunium Persicum (Black Caraway) on blood glucose indices, lipid profile, and Serum levels of nesfatin-1 in overweight or obese patients with type 2 diabetes mellitus (T2DM) had been assessed. The various clinical usages and the lack of awareness concerning the advantages and disadvantages of Bunium Persicum in patients with T2DM have made it so consistent. Based on both unadjusted and adjusted analysis models, the supplementation of 2 g of BP for 8 weeks significantly decreased FBG, HOMA-IR, and BMI and increased QUICKI in the intervention group compared with the placebo group. Also, the reduction in WC was meaningful in the unadjusted model but not significant in the adjusted model.

The human studies of the impacts of Bunium Persicum on glucose indices and lipid profiles were limited. Considering our knowledge, no study has tested the impacts of Bunium Persicum on serum levels of nesfatin-1 in these patients. Some effects of Bunium Persicum or other plants of the Apiaceae family have shown some contradictions were mentioned as follows.

The flavonoids, especially quercetin, can increase nesfatin-1 gene expression [45]. In the present trial, Bunium Persicum significantly increased the serum nesfatin-1 in the BP group; however, increasing nesfatin-1 in the BP group compared to the placebo group was not significant. The reasons are likely to be the short time of intervention and the smaller sample size. Furthermore, Kaempferol is a natural flavonol, a type of flavonoid that can up-regulate the satiety gene NUCB-2/nesfatin-1, which plays an essential role in controlling energy balance, reducing body weight, and increasing satiety [13, 46–48]. Nesfatin-1 can produce several activities, including suppressing inflammation, lipid mobilization activity, enhancement of antioxidant defense, and a regulatory role in glucose homeostasis, which can prevent and decrease the complications, development, and progression of T2DM [49–51]. Nesfatin-1 mRNA is noted in white adipose tissue, gastric mucosa, and central nervous systems (CNS) such as the hypothalamus and brainstem [52–55]. Hence, nesfatin-1 was identified in human plasma and negatively correlated with body mass index [56].

In several animal studies, caraway (Bunium Persicum and *Carum carvi*) significantly decreased FBG levels [36, 57, 58]. In contrast with our study, the caraway in obese and overweight patients over 12 weeks had no significant FBG effects [31]. It may be attributed to the sample size and form of supplementation in our intervention. A hypothesis for the possible mechanism of hypoglycemic activity of this plant may be during its main bioactive compounds. The hypoglycemic impact of limonene has been previously reported in diabetic rats by decreasing gluconeogenic enzymes’ activities, increasing the glycolytic enzymes, and stimulating insulin secretion in pancreatic β-cells [59, 60]. Cumin aldehyde in the Bunium Persicum oilseeds has significant inhibitory activity against the α-glucosidase enzyme that catalyzes the final step in the digestive process of carbohydrates. The inhibitory effect of cumin aldehyde can postpone glucose uptake and reduce hyperglycemia [26]. Also, the important proposed mechanism in the improvement of glucose indices by nesfatin-1 was the increase of glucose-induced insulin secretion by promoting Ca^2+^ influx and L-type channels [61, 62]. Thus, the observed improvements in this trial can be attributed to the increased nesfatin-1 levels by BP.

Although in this trial, lipid profile was not changed significantly, two human studies demonstrated the improvement of lipid profile by using caraway. The beneficial impacts of caraway on lipid profile, including LDL, HDL, TG, and cholesterol were observed in most animal models [19, 63].

In our study, black caraway significantly reduced weight and BMI compared to the placebo group, although waist circumference reduction was not significant. It can be attributed to the short time of intervention in our study. Results in the present study are almost consistent with previous studies. Caraway extract supplementation in overweight and obese women over 90 days decreased weight, body mass index, body fat percentage, and waist-to-hip ratio [44]. Another human study on the impacts of taking the caraway supplement and eight weeks of the aerobic exercise indicated a significant alteration in weight and BMI in the supplement-exercise group compared to other groups [64]. The proposed mechanisms in the improvement of weight, BMI, and WC may be attributed to the increased serum nesfatin-1 levels by BP. Nesfatin-1 affects energy homeostasis and decreases appetite and body weight [56, 65, 66].

The observed metabolic and clinical response to BP in T2DM patients would make this trial relevant. Although BP has some impacts on some clinical and metabolic indicators in this study, these impacts are small and need further studies with a larger sample size and longer duration and in various diseases.

The present study has several strengths in its procedure: At first, there was the initial evaluation of BP impacts in overweight or obese T2DM patients especially by evaluating nesfatin-1 levels; second, the double-blinded stratified blocked randomization design; third, evaluating dietary intake and physical activity levels and adjustment and other potential confounders; fourth, considering control group and fifth, considering multiple eligibility criteria.

However, the present study had some limitations. First, the sample size was small, second, intervention duration was short to understand the real impacts of Bunium Persicum (Black Caraway) supplementation, third, self-reporting of dietary patterns and physical activity level, fourth, failure to check the bioavailability of BP and quantitate serum levels of its ingredients, fifth, 24-h food recall is not a good index for evaluating the usual food intake sixth, failure to measure body composition and blood pressure, seventh, no evaluation of the dietary intake status of participants during the intervention period and eighth, the effect size was small.

## Conclusion

Eight-weeks supplementation of Bunium Persicum (2 g/day) in overweight or obese T2DM patients improved serum glucose indices and BMI. The use of Bunium Persicum in clinical practice needs to be further studied.

## Data Availability

The datasets used and/or analyzed during the current study are available from the corresponding author on a reasonable request.

## Abbreviations

T2DM: type 2 diabetes mellitus
BP: Bunium Persicum
BMI: body mass index
IPAQ: international physical activity questionnaire
I.C.V injection: Intracerebroventricular injection
TG: triglyceride
ELISA: enzyme-linked immunosorbent assay
TWRM-ANOVA: two-way repeated-measures analysis of covariance
